# Closed Loop Control of Penetration Depth during CO_2_ Laser Lap Welding Processes

**DOI:** 10.3390/s120811077

**Published:** 2012-08-09

**Authors:** Teresa Sibillano, Domenico Rizzi, Francesco P. Mezzapesa, Pietro Mario Lugarà, Ali Riza Konuk, Ronald Aarts, Bert Huis in 't Veld, Antonio Ancona

**Affiliations:** 1 CNR-IFN Institute for Photonics and Nanotechnologies, UOS Bari, 70126 Bari, Italy; E-Mails: rizzi@fisica.uniba.it (D.R.); francesco.mezzapesa@uniba.it (F.P.M.); lugara@fisica.uniba.it (P.M.L.); ancona@fisica.uniba.it (A.A.); 2 Laboratory of Mechanical Automation, University of Twente, 7500 AE Enschede, The Netherlands; E-Mails: A.R.Konuk@utwente.nl (A.R.K.); R.G.K.M.Aarts@utwente.nl (R.A.); A.J.HuisintVeld@utwente.nl (B.H.V.)

**Keywords:** laser welding, plasma spectroscopy, closed loop control

## Abstract

In this paper we describe a novel spectroscopic closed loop control system capable of stabilizing the penetration depth during laser welding processes by controlling the laser power. Our novel approach is to analyze the optical emission from the laser generated plasma plume above the keyhole, to calculate its electron temperature as a process-monitoring signal. Laser power has been controlled by using a quantitative relationship between the penetration depth and the plasma electron temperature. The sensor is able to correlate in real time the difference between the measured electron temperature and its reference value for the requested penetration depth. Accordingly the closed loop system adjusts the power, thus maintaining the penetration depth.

## 1. Introduction

Laser beam welding is a well established joining technique for several applications in both the aerospace [1,2] and automotive industries [3–5], by using different traditional sources, such as CO_2_ or Nd:YAG lasers. Recently, new high power fiber lasers [6,7] are receiving increasing attention in this field, thanks to their high efficiency, high power and unique beam quality. The relationship between process parameters and the final weld quality is very complex. The influence of the process parameters, including laser power, welding speed, focal point position, nozzle configuration and protection gas flow on the weld quality has been explored in recent papers by using, for example, post-process analysis of local deformation during tensile tests [8]. For these reasons, in-process monitoring is a very critical issue to avoid time-consuming post-process analysis and to obtain in-specification products at the highest production rates, by using for example CMOS camera based monitoring systems [9] or the heat radiation intensity collected from the laser irradiated area as an in-process monitoring signal [10,11]. In recent years, a lot of studies have been focused on monitoring of quality of the welded joints. Most of these methods rely on photodiode-based systems that acquire and analyze the electromagnetic emissions generated during the interaction of the laser beam with the materials [12,13]. The phenomena related to the optical emission of the laser-induced plume have been also widely investigated to develop innovative spectroscopic systems [14–16]. During the CO_2_ laser welding process, a plasma plume is generated above and inside the keyhole, which emits an intense optical radiation. Direct observation of the internal plasma is prevented by the presence of the plasma plume residing above the keyhole, as shown by Zhang *et al.* [17], while the optical emission from the plasma above the keyhole can be easily observed by suitable detectors [14,16]. Optical sensors based on fast spectrometers have the further advantage of providing a detailed analysis of the emission spectrum over a wide wavelength range.

The dynamics of the plasma plume related to change of process settings can be successfully monitored by analyzing its optical emission spectrum. Morphological changes of the spectra [18], correlation and/or anti-correlation between different spectral lines [15] and the oscillations of the plasma plume [19] have been extensively investigated in our previous works.

Successful control of the laser power during the process is one of the most significant steps towards automation, since this parameter mainly determines the energy input transferred to the workpieces. On the basis of the above described techniques, a number of closed-loop process control systems have been proposed and realized in recent years. Several works have been dedicated to the design of closed loop controllers by using the laser power as a control variable. Postma *et al.* [20] developed a closed-loop controller able to maintain full penetration in mild steel sheets during Nd:YAG laser welding. Their approach consisted in measuring the intensity of the infrared (IR) radiation coming from the weld pool. This signal has been then used as input for the closed-loop controller. However, this approach is strongly influenced by the signal level and it can suffer from instabilities due to natural perturbations of the process [21]. Differently, Bagger *et al.* [22] developed a closed-loop controller of the laser power to maintain an even seam width on the root side of the welds. They used a continuous wave (cw) CO_2_ laser source for their experiments. Here, the input signal was the integrated optical emission collected, by means of photodiodes, from the bottom of the weld. The major limitation of this system was related to the positioning of the detectors.

Several authors have demonstrated the possibilities of feedback strategies by controlling the focal-point position [23], which has significant influence on the welding process and the weld quality. The focal shift may strongly influence the laser power intensity on the samples due to non-planar work-piece or thermal focusing of the lens. Zhang *et al.* [24] developed a double-closed loop control of the focal point position for CO_2_ laser beam welding, by simultaneously using a plasma charge sensor and a photodiode sensor. The control system was able to follow the contours of the work-piece surface and to adjust the focal point position, thus compensating for the thermal focusing effect. Another system [25] exploited the light emitted during the process, together with the chromatic aberrations of the focusing optics, to determine the position of the laser beam focal spot relative to the work-piece surface. In this case, closed loop operation on the focal point position has been demonstrated over a wide range of welding conditions.

In this paper, we describe a novel spectroscopic closed loop control system capable of stabilizing the penetration depth during laser welding processes by using the laser power as the control variable. Our approach is to use the plasma electron temperature as an in process-monitoring signal. This physical quantity is estimated starting from the analysis of the optical radiation emitted by the plasma plume above the keyhole. The laser power has been controlled exploiting the quantitative relationship between the penetration depth and the plasma electron temperature. Lap welding of stainless steel sheets has been carried out with a cw CO_2_ laser. The sensor measures the difference between the real time value of the electron temperature and a reference value, corresponding to the requested penetration depth. Accordingly, the closed-loop controller adjusts the laser power in order to reduce this temperature difference and maintain the desired penetration depth. The main objective of this system is to reduce the process waste due to incomplete or excessive weld penetrations, thus increasing the production rate and lowering the costs. Several aspects of the welding process have been also explored in order to evaluate the robustness of the closed loop controller. In particular we have investigated how rapidly any perturbation of the incident laser power induces an appreciable change of both the penetration depth and the plasma electron temperature and, correspondingly, how fast is the response of the designed control system.

## 2. Experimental Procedure

### 2.1. Experimental Details

**Figure 1.**
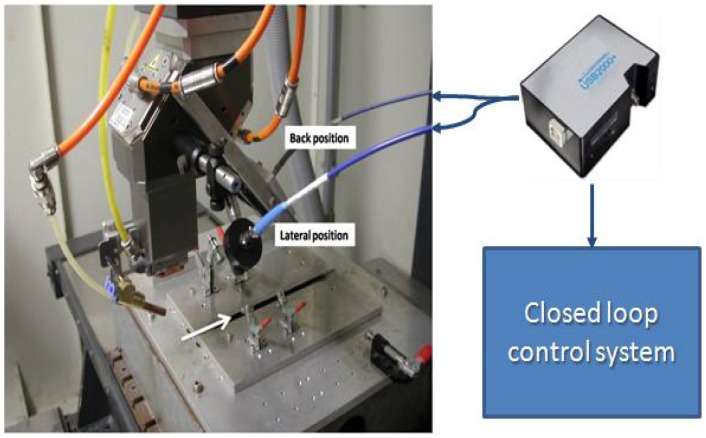
Experimental set-up.

Lap weldings were carried out with a CO_2_ laser source delivering a maximum output power of 2,500 W in cw regime, focused onto the workpiece surface through a parabolic focusing mirror of 200 mm focal length. For the welding trials, overlap welds have been realized with 1 mm-thick on top of 2 mm-thick AISI304 stainless steel plates. The welding speed has been kept constant at 50 mm/s for all the experiments, as well as the beam focus position on the top surface of the sample. The argon gas has been provided from the top side of the weld at a flow rate of 60 L/min and a nozzle stand-off distance of 6 mm, as depicted in Figure 1. Only the laser power has been changed during the experiments, from 800 W to 1,700 W, corresponding to a heat input ranging from 16 J/mm to 36 J/mm [26], to obtain different penetration depth values.

The plasma optical emission has been collected by a 6-mm focal length collimator which was placed at an angle of about 60 degrees with respect to the laser beam axis. The collected radiation was sent through a 200 μm-core fiber to the 10 μm entrance slit of an Ocean Optics USB2000+ miniature spectrometer equipped with a holographic grating having a groove density of 1,800 L/mm, and a 2,048 pixel CCD detector array. Despite of the limited spectral bandwidth, from 400 nm to 530 nm, the spectrometer has been designed in order to have a relatively high spectral resolution of 0.12 nm which is necessary to resolve the huge amount of spectral lines composing a typical CO_2_ laser welding plasma emission spectrum. Previous studies have indeed shown that, in case of CO_2_ laser welding of stainless steel, most of the plasma optical emission lines useful for the electron temperature calculation are found in this spectral range [26,27].

To measure the bead profiles the samples were sectioned, polished and macroscopically etched to reveal the structures of interest. Metallographic analyses of the welded joints cross sections have been carried out in order to measure the penetration depth obtained under several operating condition by using a stereo microscope equipped with a CCD camera and suitable software for image capture and analysis.

### 2.2. Electron Temperature Calculation

The AISI304 steel alloy is mainly composed of iron, chromium and manganese. The plasma emission spectra acquired during our experiments showed, in the investigated spectral range, a dense discrete contribution consisting of hundreds of emission lines, belonging to the aforementioned chemical species [26]. Due to the relatively low laser power employed (from 800 to 1,700 W), the plasma was weakly ionized, thus most of the spectral lines were ascribed to iron, chromium and manganese in their atomic excited states: Fe(I), Cr(I), Mn(I), respectively.

The high resolution of our spectrometer allowed us to spectrally resolve and catalogue the chemical species present in the plasma, with the help of the NIST atomic spectra database [28]. From our previous studies [26,27] we selected the most suitable pair of lines, to be used for the electron temperature calculations.

This physical quantity has been calculated using the line intensity ratio method, according to the following equation [29], after verifying that the hypothesis of Local Thermal Equilibrium was valid:

(1) $${\text{T}}_{\text{e}}=\frac{{\text{E}}_{1}-{\text{E}}_{2}}{\text{k}\ln\left(\frac{{\text{I}}_{1}\lambda_{1}{\text{A}}_{2{\text{g}}_{2}}}{{\text{I}}_{2}\lambda_{2}{\text{A}}_{1{\text{g}}_{1}}}\right)}$$

In this equation, the labels 1 and 2 refer to two spectral lines of the same element and I_i_, λ_i_, g_i_, A_i_ and E_i_ (i = 1, 2) represent the line intensity, wavelength, statistical weight, transition probability and the energy of the excited state, respectively. T_e_ and k are the electron temperature and the Boltzmann constant, respectively.

The welding plasma emission spectra have been acquired by using the spectrometer at its highest rate of 1 kHz, corresponding to an acquisition time of 1 ms for each spectrum. The acquired data were processed in real-time by a Labview program performing the identification of the pre-selected couple of lines (see references [26,27] for more details), their intensity measurement and the plasma electron temperature computation and visualization at the user interface. The overall sampling time of the Labview code, including the acquisition time, was found to be around 5 ms, resulting in a maximum signal sampling rate of about 200 Hz.

## 3. Closed Loop Design and Configuration

**Figure 2.**
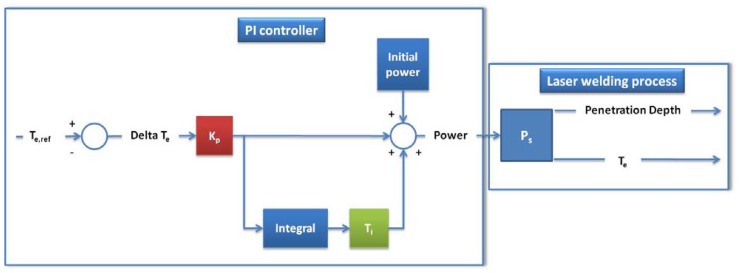
Controller layout.

**Figure 3.**
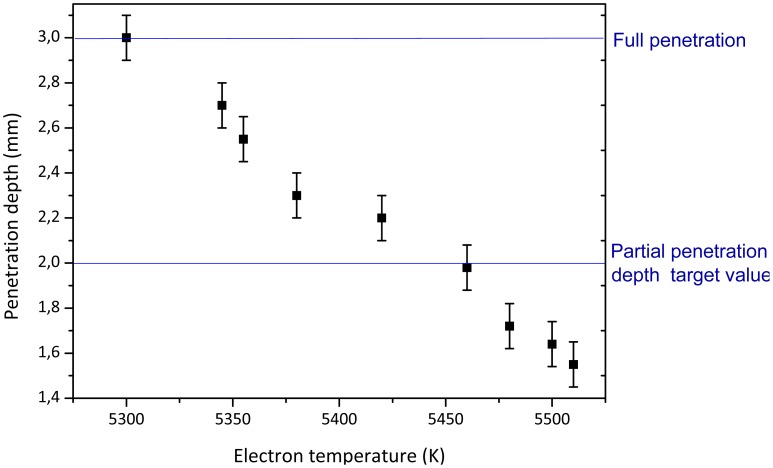
Characterization curve of penetration depth as a function of the Fe(I) electron temperature, at a welding speed of 50 mm/s.

Figure 2 shows an overview of the controller operation. The electron temperature signal was used by the controller to stabilize the penetration depth at the desired value by adjusting the laser power. The core of the controller is a Proportional-Integral (PI) algorithm, based on the following equation:

(1) $$\text{C}(\text{s})={\text{K}}_{\text{p}}\left(1+\frac{1}{{\text{sT}}_{\text{i}}}\right)$$

in which K_p_ and T_i_ are constant parameters that denote controller gain and integral time constant parameters, respectively, and variable *s* indicates that the equation is expressed in the Laplace domain. The electron temperature set point T_e,ref_, for a desired penetration, has been determined according to the relation found in the preliminary welding tests and expressed in Figure 3. Like the P-only controller, the PI algorithm computes a controller output from its input. In our case the controller output was an analogic voltage signal (0–10 V) linearly related to the laser power level (0–2,500 W). The input signal was the difference ΔT_e_ between the current electron temperature signal and its reference value. The computed output from the PI algorithm depends on the controller tuning parameters and the controller's input. The integral action ensures that the PI controller eliminates an offset, which is not possible with a P-only controller. To improve the controller performance at the start of the weld an initial laser power based on previous experiments is added to the controller output. Thus, PI controllers provide a balance of complexity and capability that makes them a widely used algorithm in process control applications.

## 4. Results and Discussion

### 4.1. Characterization Tests

Preliminary welding tests aimed at defining a relationship between the penetration depth and the electron temperature. For this purpose, several welding trials have been performed by dynamically changing the incident laser power and by acquiring the plasma emission spectra at the fastest achievable rate.

Results demonstrate that the electron temperature is a consistent physical quantity able to identify a dynamic change of the penetration depth caused by the varying laser power, as showed in Figure 3. Here, the penetration depth is measured starting from the surface of the work-piece where the laser beam impinges, *i.e.*, on top of the 1-mm-thick steel sheet overlapped onto the 2-mm-thick specimen. Therefore, a 2-mm-deep weld corresponds to a full penetration of the top sheet and half a penetration of the bottom sheet, while a 3-mm penetration depth indicates a fully penetrated lap joint all-through the two specimens. A detailed description of the characterization procedure is reported in our previous works [11].

The singular behavior of the Fe(I) electron temperature is worth noting: all experiments showed that the plasma temperature apparently decreases with the laser power, thus resulting to be inversely proportional to the penetration depth [11,12]. This trend of the signal has to be ascribed to the position of the light collecting system with respect to the plasma plume above the keyhole. In our experiments, the angle of view of the collimator was fixed at around 60° degrees with respect of the beam direction, and it mainly pointed on top of the keyhole. Thus, for deeper penetrations, only the radiation emitted by the outer and colder shell of the plume was collected and lower temperature values were thus measured as far as a higher penetration depth was achieved.

### 4.2. Closed Loop Results

Two series of tests were conducted to investigate the performance of the controller in response to a linear change of the laser power. In the first series, a 2 mm penetration depth has been chosen as target value. Preliminary welding tests, described in Section 2.2, have been carried out to find the laser power P necessary to obtain a joint penetration depth of 2 mm over 3 mm full sample thickness, at a fixed value of the welding speed. The optimal process parameters have been found to be a laser power of 1,200 W at a welding speed of 50 mm/s. The electron temperature was calculated using two neutral iron (Fe(I)) emission lines at 421.91 nm and 517.09 nm respectively. As a result of the characterization experiments, the corresponding Fe(I) electron temperature set-point has been found to be 5,460 K, as shown in Figure 3. The optimization of the controller parameters K_p_ and T_i_ have been carried out performing several welds at predetermined laser process parameters, looking for the quickest and steadiest response of the controller performances. As a result, the K_p_ and T_i_ values were set to −2 W/K and 150 ms, respectively. It is worth noting that a negative value for K_p_ is only related to the inverse proportionality between the laser power and the electron temperature signals.

**Figure 4.**
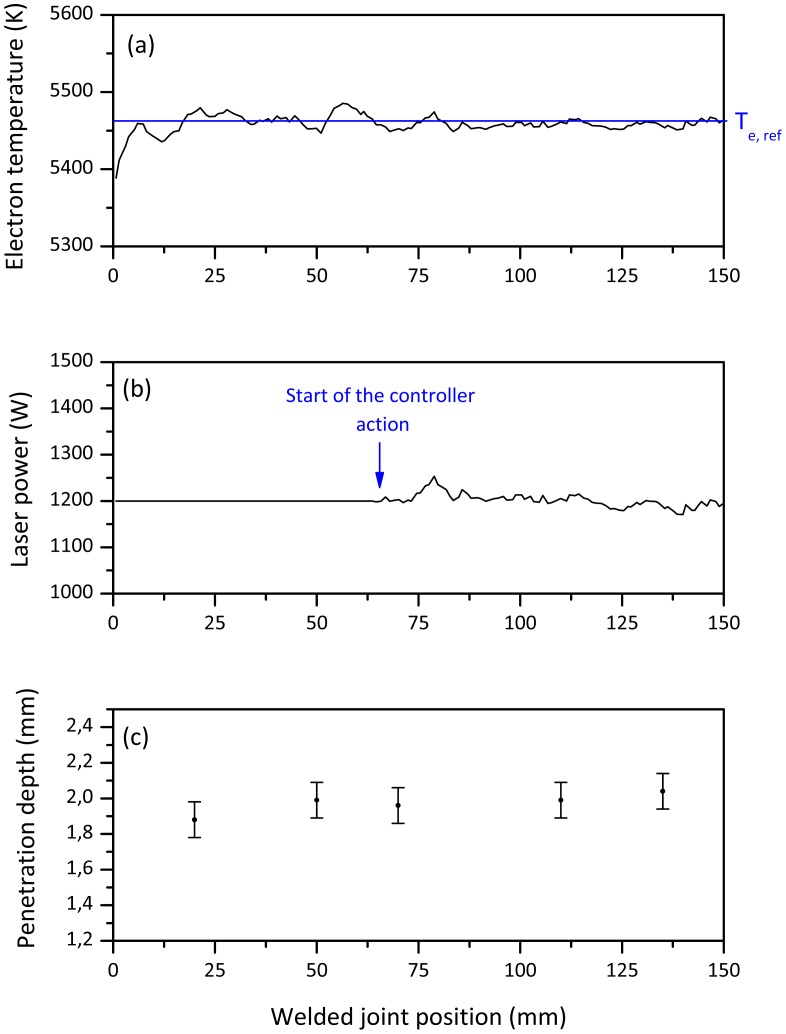
(**a**) Electron temperature signal behavior. Controller parameters: T_e,ref_ = 5,460 K corresponding to a penetration depth of 2 mm; K_p_ = −2 W/K; T_i_ = 150 ms. Initial laser power = 1,200 W. (**b**) Laser power measured by fast power sensor. (**c**) Penetration depth measurements.

The experiments have been designed such that the controller drives the early stage of the weld at a constant predetermined initial laser power value. After that time interval, the controller starts its action by comparing the measured electron temperature with the expected one at the desired penetration depth. Figure 4(a,b) shows the electron temperature and the laser power measured by the sensor respectively for a welding test performed by setting a starting power level equal to the optimal value. The transverse cross sections (Figure 4(c)) in different point of the welded joint confirmed a steady average joint depth of around 2 mm.

**Figure 5.**
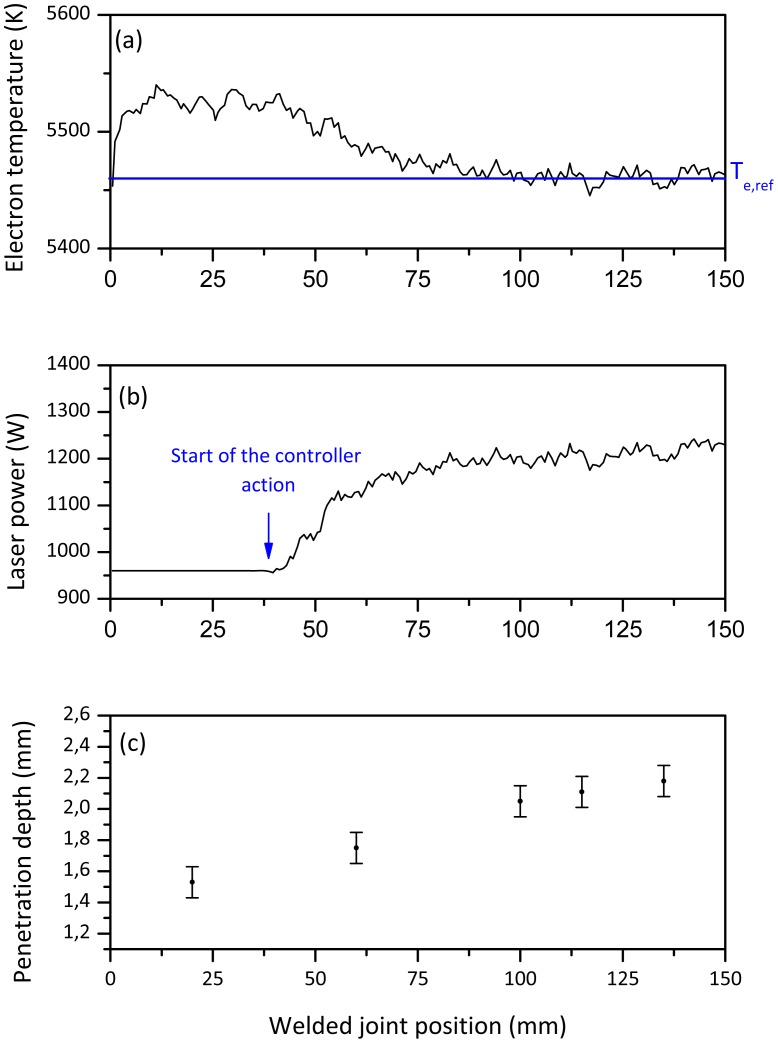
(**a**) Electron temperature signal behavior. Controller parameters: T_e,ref_ = 5,460 K corresponding to a penetration depth of 2 mm; K_p_ = −2 W/K; T_i_ = 150 ms. Initial laser power = 960 W. (**b**) Laser power measured by fast power sensor. (**c**) Penetration depth measurements.

The in-process monitoring results, under feedback control, are demonstrated more clearly in Figure 5. The initial laser power has been set at 960 W, 20% lower than the optimal set point, in order to test the ability and the response speed of the controller to reach the target penetration depth when it is activated. As described in Section 2.2, a higher value of the electron temperature was measured for a lower value of incident power, resulting in a lack of penetration. Figure 5(b) shows how the control system was able to rapidly increase the laser power from 960 W to 1,200 W, while the electron temperature almost linearly decreased from the initial value to its set point. Here, a stable penetration depth, very close to the target value, was achieved, as evidenced by the transverse cross sections measurements showed in Figure 5(c).

**Figure 6.**
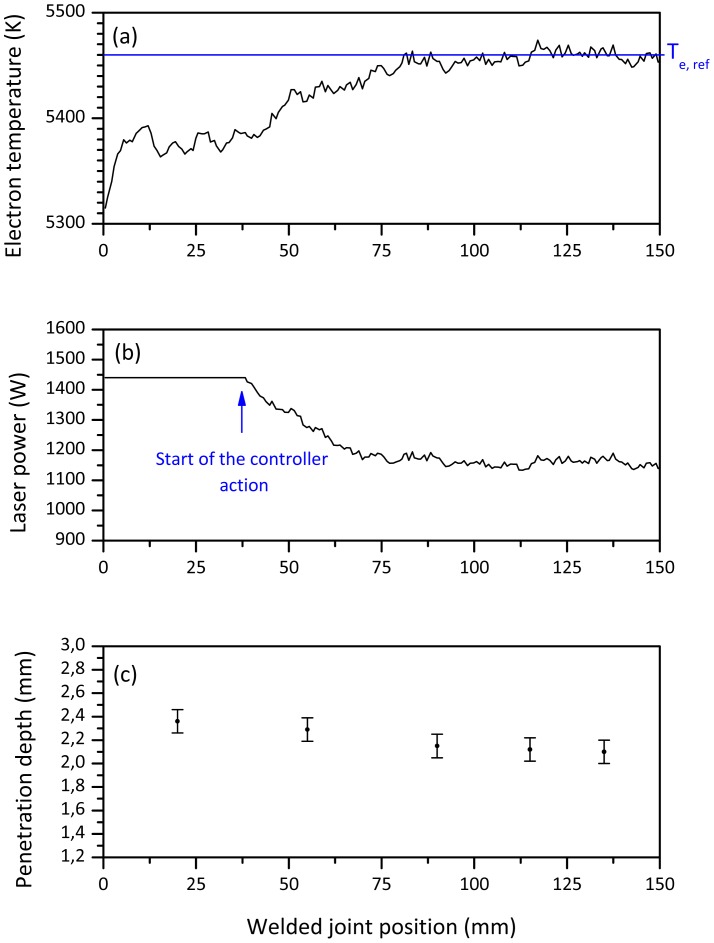
(**a**) Electron temperature signal behavior. Controller parameters: T_e,ref_ = 5,460 K corresponding to a penetration depth of 2 mm; K_p_ = −2 W/K; T_i_ = 150 ms. Initial laser power = 1,440 W. (**b**) Laser power measured by fast power sensor. (**c**) Penetration depth measurements.

Finally, the starting laser power was set at 1,440 W, 20% higher than the optimal value, thus producing a higher penetration depth as shown in Figure 6(a,b). As soon as the controller was activated, the laser power started to decrease and, consequently, the measured electron temperature signal began to rise until its set point was achieved, corresponding to the optimized laser power value. Also in this case, the results of macrographs, plotted in Figure 6(c), showed that the penetration gradually approached and stabilized to its target value of 2 mm.

In the second series of experiments, we tested the reliability of the sensor by setting a different value for the target penetration. Here, a joint depth of 3 mm has been chosen as target value, corresponding to a full penetration through the two overlapped steel sheets. Such a condition was achieved at a laser power of 1,800 W and a welding speed of 50 mm/s, corresponding to an electron temperature set point of 5,290 K.

**Figure 7.**
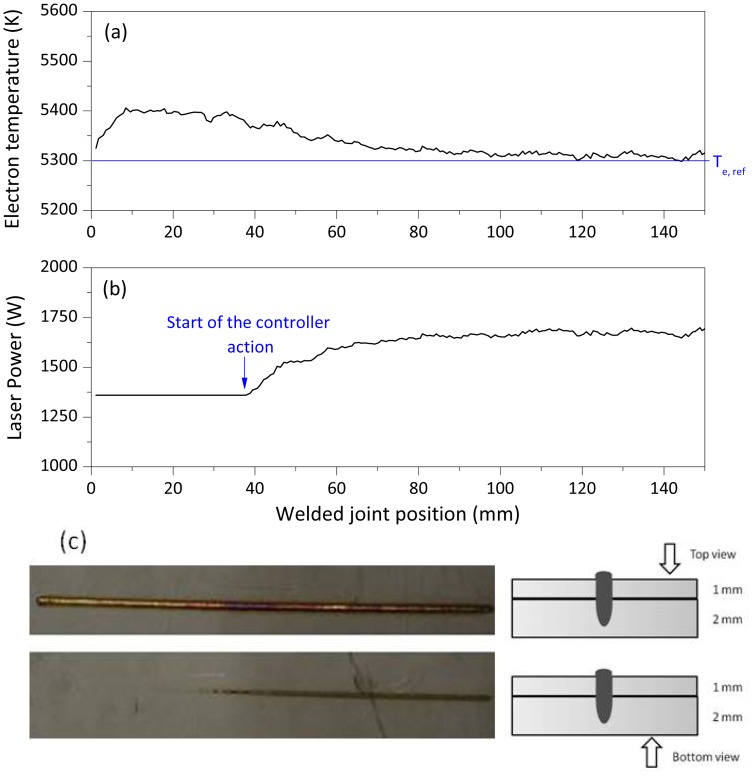
(**a**) Electron temperature signal behavior. Controller parameters: T_e,ref_ = 5,300 K corresponding to a (full) penetration depth of 3 mm; K_p_ = −2 W/K; T_i_ = 150 ms. Initial laser power = 1,450 W. (**b**) Laser power measured by fast power sensor. (**c**) Top and bottom view of the welded joint.

Figure 7 shows the results obtained by setting a starting value for the laser power 20% lower than 1,800 W. When activated, the controller adjusted the analog voltage signal driving the laser power, according to how far the measured electron temperature was from its set point. A correction of the penetration depth was obtained after around 1 second, as shown by the visual inspection of the rear side of the joint. Here, a fully penetrated weld has been observed in the last part of the seam, when the controller was activated thus driving the laser power to its optimal value of about 1,800 W.

Further experiments have been performed where the laser power has been varied over a wide range and with different target penetration depths. The reliability of our closed loop control system was confirmed with quite fast response times of around a few ms under these different conditions.

## 4. Conclusions

In this work, an efficient nonintrusive closed loop control system for CO_2_ laser welding, based on a spectroscopic approach, has been successfully demonstrated. The lap welding process of stainless steel sheets has been characterized to obtain a relationship between the plasma electron temperature and the penetration depth. Once activated, our control system is able to adjust the laser power in order to reach and hold a required penetration depth through the real time measurement of the welding plasma electron temperature. The developed control system is potentially adaptable to different experimental conditions, and key controller parameters can be readily modified in order to ensure successful operation when welding parameters are changed. Precise calibration is required to adapt the controller to each specific experimental configuration (laser type, material and penetration depth). Further investigations will be addressed to investigate the response of the system to a change of welding speed or the weld geometry, e.g., butt-welding.
